# 2-Oxoesters: A Novel Class of Potent and Selective Inhibitors of Cytosolic Group IVA Phospholipase A_2_

**DOI:** 10.1038/s41598-017-07330-5

**Published:** 2017-08-01

**Authors:** Maroula G. Kokotou, Gerasimia Galiatsatou, Victoria Magrioti, Giorgos Koutoulogenis, Efrosini Barbayianni, Dimitris Limnios, Varnavas D. Mouchlis, Banita Satpathy, Aaron Navratil, Edward A. Dennis, George Kokotos

**Affiliations:** 1 Laboratory of Organic Chemistry, Department of Chemistry, National and Kapodistrian University of Athens, Panepistimiopolis, Athens 15771 Greece; 2 Department of Chemistry and Biochemistry and Department of Pharmacology, School of Medicine, University of California, San Diego, La Jolla, California 92093-0601 USA

## Abstract

Cytosolic phospholipase A_2_ (GIVA cPLA_2_) is the only PLA_2_ that exhibits a marked preference for hydrolysis of arachidonic acid containing phospholipid substrates releasing free arachidonic acid and lysophospholipids and giving rise to the generation of diverse lipid mediators involved in inflammatory conditions. Thus, the development of potent and selective GIVA cPLA_2_ inhibitors is of great importance. We have developed a novel class of such inhibitors based on the 2-oxoester functionality. This functionality in combination with a long aliphatic chain or a chain carrying an appropriate aromatic system, such as the biphenyl system, and a free carboxyl group leads to highly potent and selective GIVA cPLA_2_ inhibitors (*X*_I_(50) values 0.00007–0.00008) and docking studies aid in understanding this selectivity. A methyl 2-oxoester, with a short chain carrying a naphthalene ring, was found to preferentially inhibit the other major intracellular PLA_2_, the calcium-independent PLA_2_. In RAW264.7 macrophages, treatment with the most potent 2-oxoester GIVA cPLA_2_ inhibitor resulted in over 50% decrease in KLA-elicited prostaglandin D_2_ production. The novel, highly potent and selective GIVA cPLA_2_ inhibitors provide excellent tools for the study of the role of the enzyme and could contribute to the development of novel therapeutic agents for the treatment of inflammatory diseases.

## Introduction

In mammals, the phospholipase A_2_ (PLA_2_) superfamily consists of six types of diverse enzymes: GIV PLA_2_ [cytosolic PLA_2_ (cPLA_2_)], GVI PLA_2_ [calcium-independent PLA_2_ (iPLA_2_)], several groups of secreted PLA_2_ (sPLA_2_), two groups of platelet-activating factor-acetylhydrolases PLA_2_ (PAF-AHs), GXV PLA_2_ (lysosomal PLA_2_), and GXVI PLA_2_ (adipose PLA_2_)^1^. Among all these enzymes, cPLA_2_ is the only PLA_2_ that exhibits a marked preference for hydrolysis of arachidonic acid at the *sn*-2 position of phospholipid substrates^2^. The activation of cPLA_2_ results in the production of arachidonic acid and lysophospholipids giving rise to the generation of diverse lipid mediators, such as leukotrienes, prostaglandins, lysophosphatidic acid etc^3^. Since many of them are involved in the response to inflammation, the regulation of cPLA_2_ is of great importance in chronic inflammatory conditions^1, 4^. In a recent review article, Leslie has summarized the physiological function and the role of cPLA_2_ in diseases^5^. The most recent studies on inherited GIVA cPLA_2_ deficiency demonstrate the fundamental role of this enzyme in eicosanoid formation and cellular responses in human circulation^6^.

It was thirty years ago, when the first cytosolic PLA_2_ activity (now attributed to GIVA cPLA_2_ or cPLA_2_ *α*) was reported in human neutrophils and platelets^7, 8^. The purification, sequence, and cloning of the first human cPLA_2_ was reported in 1991^9, 10^. GIVA cPLA_2_ contains 749 amino acids, is an 85 kDa protein, and consists of an N-terminal C2 domain and a C-terminal catalytic domain. The crystal structure of GIVA cPLA_2_ was solved by Dessen *et al*. in 1999^11^. The catalytic domain of GIVA cPLA_2_ utilizes an unusual catalytic dyad, Ser-228/Asp-549, located in the α/β hydrolase domain, to catalyze the hydrolysis of the substrate phospholipid^12, 13^.

**Figure 1:**
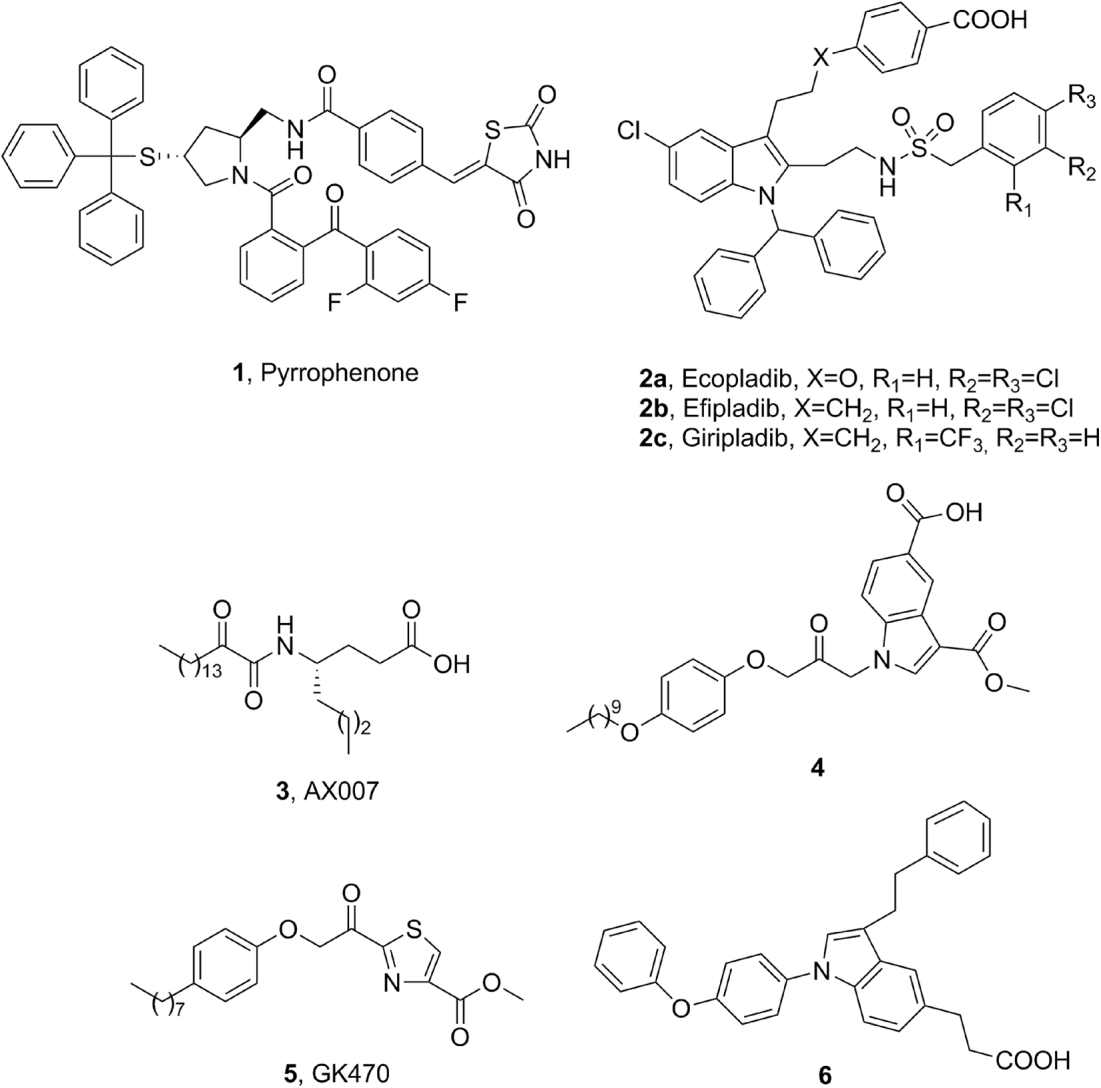
Common inhibitors of phospholipases A_2_.

The diverse bioactive lipids produced by the cPLA_2_ activity regulate normal physiological processes and disease pathogenesis, and as a consequence, great attention has been given to the development of selective GIVA cPLA_2_ inhibitors. The structural diversity of the synthetic inhibitors is summarized in a number of review articles^1, 14–16^. The first synthetic inhibitor of GIVA cPLA_2_ was an arachidonic acid derivative, arachidonoyl trifluoromethyl ketone, containing an activated carbonyl functionality^17^. Shionogi developed a series of pyrrolidine-based inhibitors, including pyrrophenone (**1**, Fig. 1), following a high throughput screening approach^18, 19^. Wyeth has expended major efforts to develop novel indole-based inhibitors, for example, ecopladib (**2a**, Fig. 1), efipladib (**2b**, Fig. 1) and giripladib (**2c**, Fig. 1) as novel therapeutics for inflammatory diseases^20–23^. Giripladib was the most promising among them as it was advanced into a Phase II clinical trial for osteoarthritis, however in 2007 the trial was terminated due to gastrointestinal side effects^24^. A structurally related GIVA cPLA_2_ inhibitor is currently on phase I/II clinical study in healthy volunteers and patients with moderate to severe dermatitis and the estimated date of completion is June 2017^25^. Our groups have designed and developed long chain 2-oxoamides based on unnatural amino acids, for example compound **3**, as GIVA cPLA_2_ inhibitors^26–31^. Lehr and coworkers studied a variety of activated carbonyl-based indol-1-yl-propan-2-ones, for example compound **4** (Fig. 1) containing a variety of substituents on the heterocyclic ring to optimize the enzyme-inhibitor binding^32–36^. Recently, we have reported the new thiazolyl ketone GK470^37^ (**5**, Fig. 1) as a GIVA cPLA_2_ inhibitor, while Tomoo and colleagues demonstrated a new series of indole-based inhibitors, such as inhibitor **6** ^38^.

To fully understand the role that each particular PLA_2_ type plays in physiological and pathological conditions, and to develop new candidates for the treatment of various inflammatory diseases, potent and selective GIVA cPLA_2_ inhibitors are needed. In this work, we present a novel class of potent and selective GIVA cPLA_2_ inhibitors and our studies on their synthesis and study of their *in vitro* inhibitory potency and selectivity.

## Results

### Design and synthesis of inhibitors

**Figure 2:**
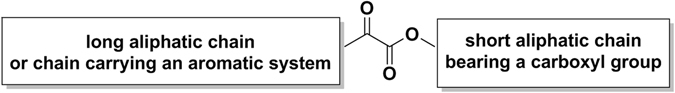
Design of 2-oxoesters.

Upon activation by intracellular calcium binding to the C2 domain of GIVA cPLA_2_, the enzyme is translocated to the surface of the phospholipid membrane where it extracts a single phospholipid substrate into the active site^39, 40^. Then, the catalytic active site serine attacks the ester bond of the phospholipid substrate initiating the hydrolysis step. A number of the existing potent GIVA cPLA_2_ inhibitors, for example arachidonoyl trifluoromethyl ketone^17^, 2-oxoamides^26–31^, indolyl-propanones^32–36^, thiazolyl ketones^37^ contain an activated carbonyl group able to interact with the active site serine. In our quest for novel potent and selective GIVA cPLA_2_ inhibitors, we envisaged that the 2-oxoester (or α-keto ester) functionality could serve as such an activated carbonyl group. In 1990, it was demonstrated that α-keto ester derivatives of *N*-protected amino acids and peptides inhibit serine and cysteine proteinases^41^, while peptidyl α-keto esters inhibit the serine proteases porcine pancreatic elastase and human neutrophil elastase^42^. Later on, various peptide α-keto-esters and α-keto acids were reported as inhibitors of calpains and other cysteine proteases^43^ and of hepatitis C virus NS3 protease^44^. It is quite clear that a potential GIVA cPLA_2_ inhibitor, in addition to a functionality targeting the active site serine, should contain a lipophilic chain able to mimic the interactions of the substrate arachidonoyl chain with the lipophilic binding site of the enzyme. In addition, a free carboxyl group may contribute significantly to the overall binding of the inhibitor to the enzyme. As we have proposed in the past^26^, and according to the results of our mechanistic studies using a combination of hydrogen-deuterium exchange mass spectrometry with molecular dynamics simulations^31^, such a carboxyl group may interact with the side chain of the enzyme residue Arg-200. Taken together, we designed compounds containing a 2-oxoester functionality, a lipophilic chain and a free carboxyl group (Fig. 2).

**Figure 3:**
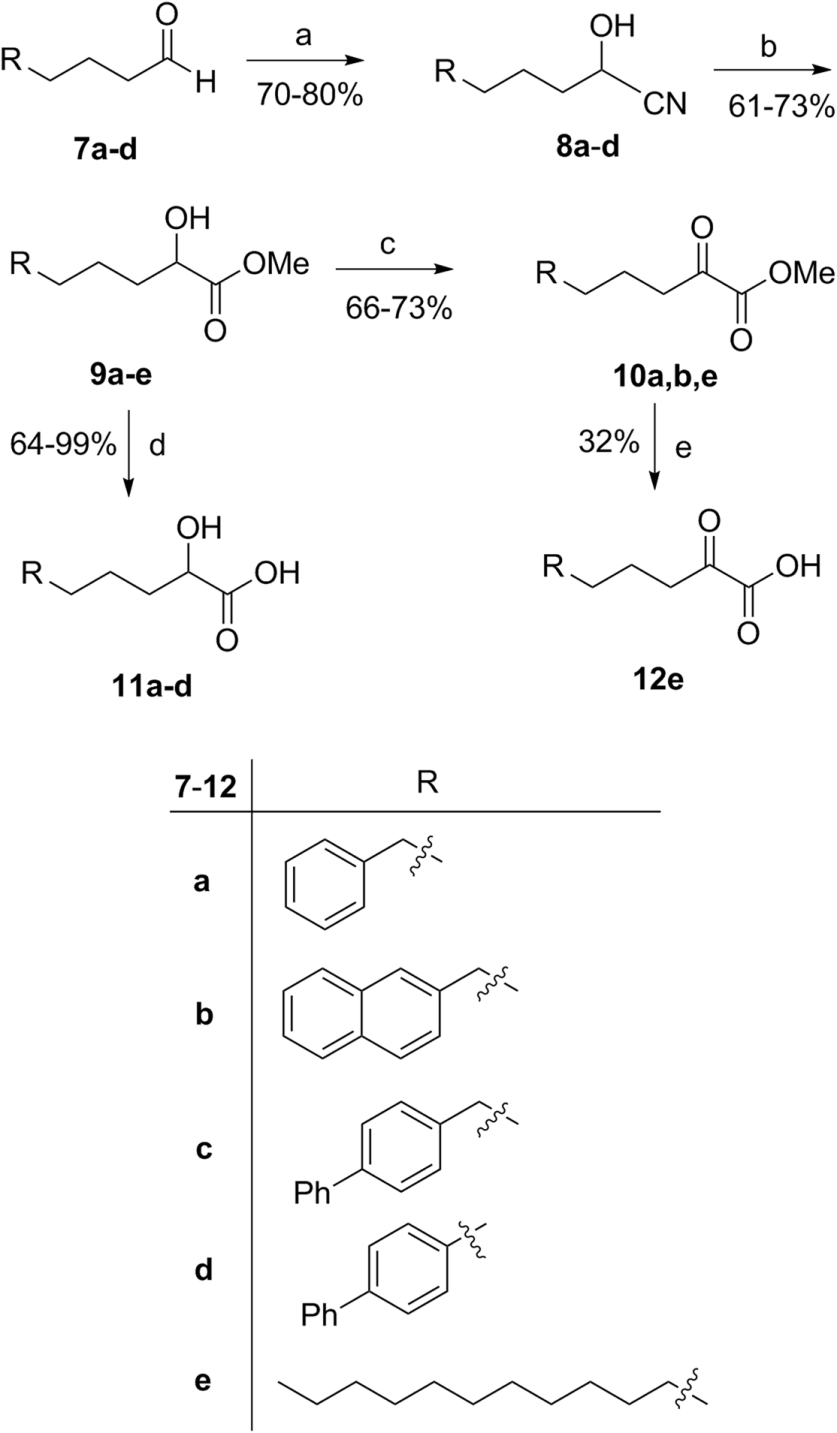
Synthesis of 2-hydroxy acids and 2-oxoacids. (**a**) (i) aq. sol. NaHSO_3_, CH_2_Cl_2_, (ii) KCN, H_2_O; (**b**) 4 N HCl/CH_3_OH; (**c**) Dess-Martin periodinane reagent, dry CH_2_Cl_2_; (**d**) NaOH 1 N, CH_3_OH; (**e**) 20% aq. sol. Cs_2_CO_3_, CH_3_OH.

A variety of 2-hydroxy acids, required for the synthesis of 2-oxoesters, were synthesized as described in Fig. 3. Aldehydes **7a**-**d** were converted into cyanohydrins **8a**-**d** and consequently to 2-hydroxy methyl esters **9a**-**d** by treatment with HCl in methanol. 2-Hydroxy acids **11a**-**d** were obtained by alkaline hydrolysis of **9a**-**d**. In addition, 2-hydroxy methyl esters **9a**,**b**,**e** were oxidized to the corresponding 2-oxoesters **10a**,**b**,**e** (Fig. 3). Free 2-oxohexadecanoic acid **12e** was synthesized by mild alkaline hydrolysis of **10e** using aqueous Cs_2_CO_3_ in methanol, as depicted in Fig. 3.

**Figure 4:**
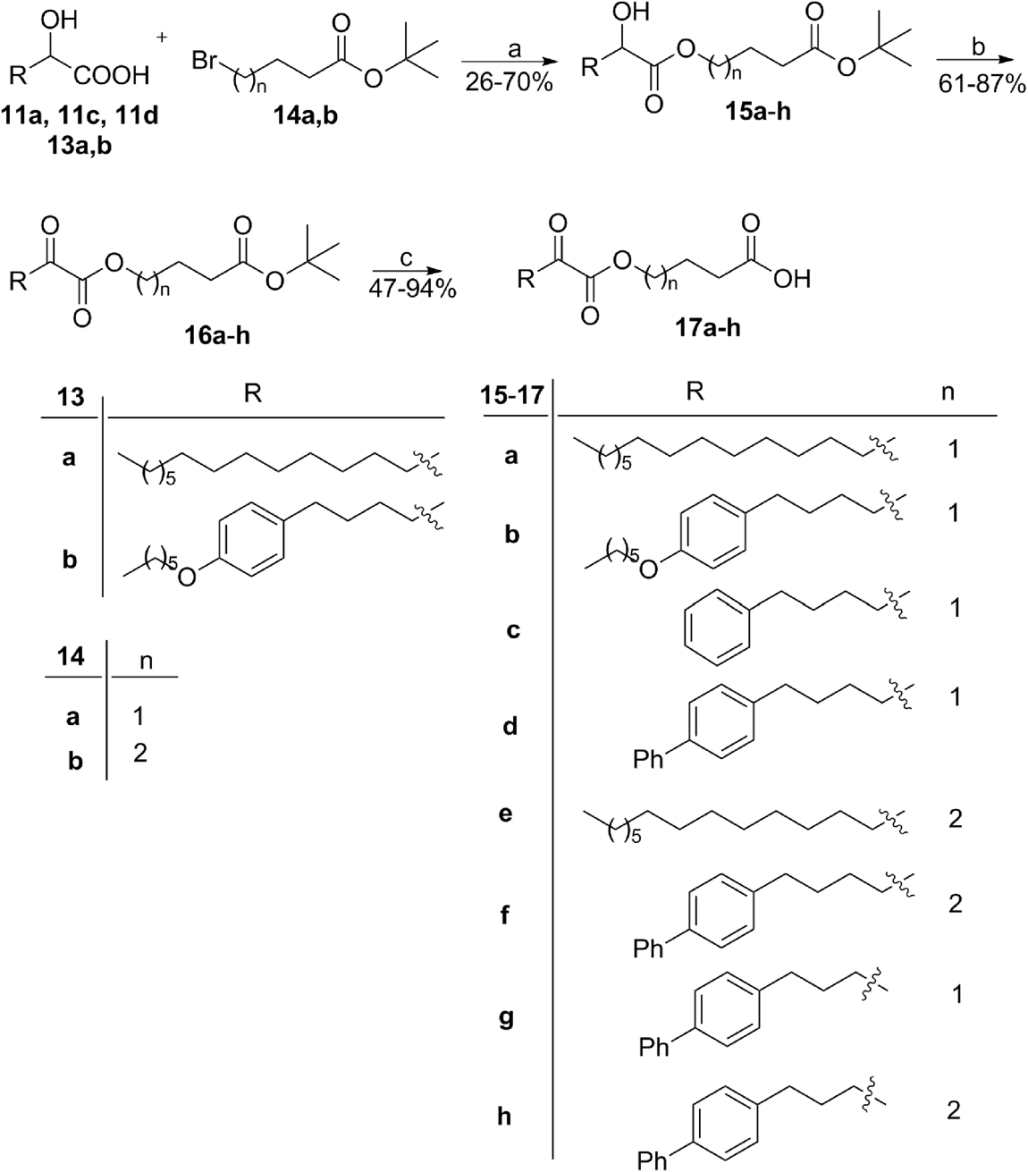
Synthesis of 2-oxoesters. (**a**) i. 20% aq. sol. Cs_2_CO_3_, THF, H_2_O, ii. Br(CH_2_)_n_CH_2_CH_2_COOBu^t^, DMF, reflux overnight; (**b**) Dess-Martin periodinane reagent, dry CH_2_Cl_2_; (**c**) 50% CF_3_COOH in CH_2_Cl_2_.

The general route for the synthesis of the designed 2-oxoesters carrying a free carboxyl group is quite straightforward and is depicted in Fig. 4. The key-step was the reaction between the cesium salt of the appropriate 2-hydroxy acids **11a, 11c, 11d and 13a,b** with omega-bromo esters **14a**,**b**. The resulting 2-hydroxy esters **15a**-**h** ware then oxidized to the corresponding 2-oxoesters **16a**-**h** using preferably the Dess-Martin periodinane reagent^45^. Removal of the *tert*-butyl ester protecting group under acidic conditions led to the target compounds **17a**-**h**.

**Figure 5:**
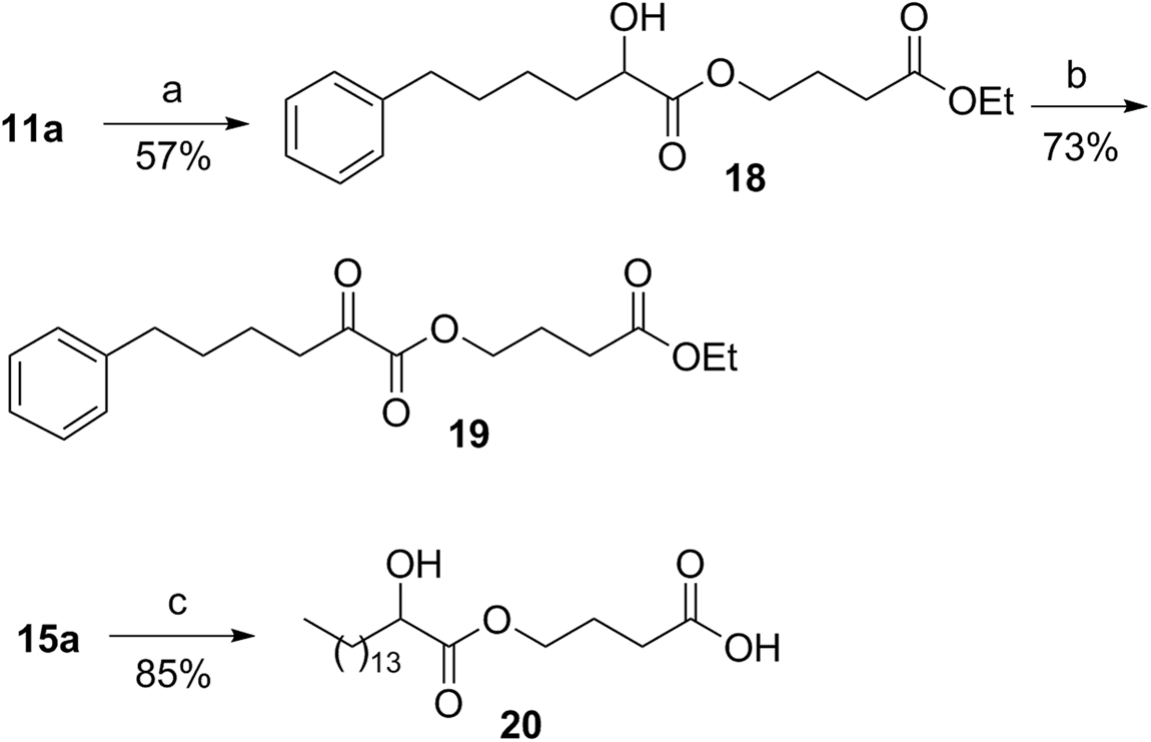
Synthesis of compounds **19** and **20**. (**a**) i. 20% aq. sol. Cs_2_CO_3_, THF, H_2_O, ii. BrCH_2_CH_2_CH_2_COOEt, DMF, reflux overnight; (**b**) Dess-Martin periodinane reagent, dry CH_2_Cl_2_; (**c**) 50% CF_3_COOH in CH_2_Cl_2_.

2-Oxoester **19** carrying an ethyl ester group and 2-hydroxyester **20** carrying a free carboxyl group were synthesized as depicted in Fig. 5.

### *In vitro* inhibition of GIVA cPLA_2_, GVIA iPLA_2_ and GV sPLA_2_

All synthesized 2-oxoesters were tested for their *in vitro* activity on recombinant human GIVA cPLA_2_ using mixed micelle assays. In addition, their selectivity over human GVIA iPLA_2_ and GV sPLA_2_ was also studied using group specific mixed micelle assays_._ The activity of these PLA_2_s was tested on mixed-micelles containing 100 µM PAPC and 400 µM Triton-X.

The *in vitro* inhibition of human GIVA cPLA_2_, GVIA iPLA_2_ and GV sPLA_2_ was carried out using previously described mixed micelle-based assays^27, 28, 30^. The inhibition results are presented in Table 1, either as percent inhibition or as *X*_I_(50) values. At first, the percent of inhibition for each PLA_2_ enzyme at 0.091 mole fraction of each inhibitor was determined. Then, the *X*_I_(50) values were measured for compounds that displayed greater than 95% inhibition of GIVA cPLA_2_. The *X*_I_(50) is the mole fraction of the inhibitor in the total substrate interface required to inhibit the enzyme activity by 50%.

**Table 1:** *In vitro* inhibitory potency and selectivity of 2-oxoesters.

Entry	No	Structure	GIVA cPLA_2_		GVIA iPLA_2_		GV sPLA_2_	ClogP
			% Inhibition^a^	*X*_I_(50)	% Inhibition^a^	*X*_I_(50)	% Inhibition^a^	
1	**10e**		68.2 ± 2.7		69.4 ± 12.2		27.5 ± 0.9	6.51
2	**12e**		78.4 ± 3.5		<25		<25	5.63
3	**17a**		>95	0.00008 ± 0.00001	<25		<25	6.76
4	**20**		<25		<25		41.0 ± 0.2	6.82
5	**17b**		>95	0.00289 ± 0.00043	<25		52.6 ± 5.3	5.46
6	**16c**		<25		<25		<25	4.58
7	**19**		27.3 ± 4.8		<25		<25	3.87
8	**17d**		>95	0.00068 ± 0.00007	<25		<25	4.78
9	**17e**		> 95	0.00007 ± 0.00001	25		<25	6.68
10	**17f**		> 95	0.000078 ± 0.00001	65 ± 3.4		<25	4.70
11	**17g**		> 95	0.0065 ± 0.002	84 ± 1.5		<25	4.25
12	**17h**		> 95	0.0010 ± 0.0003	94 ± 1.4		<25	4.17
13	**10a**		<25		72 ± 4		<25	
14	**10b**		55 ± 4.0		> 95	0.0052 ± 0.0007	<25	3.81
15	**4**		>95	0.00008 ± 0.000005^c^				8.50

^a^% Inhibition at 0.091 mole fraction of each inhibitor. ^b^IC_50_ 4.3 nM in a vesicle assay^32^.

**Figure 6:**
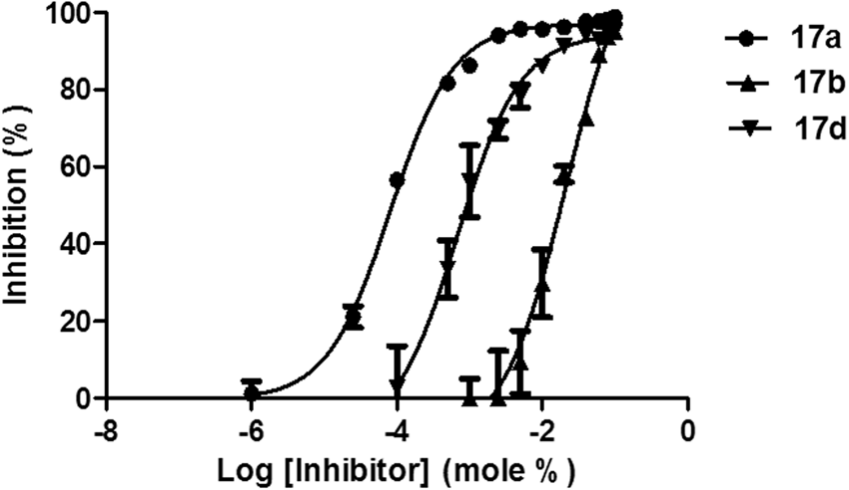
Inhibition curves for **17a**, **17b** and **17d**. The curves were generated using GraphPad Prism with a nonlinear regression targeted at symmetrical sigmoidal curves based on plots of % inhibition versus log(inhibitor concentration). The reported *X*_I_(50) values were calculated from the resultant plots.

Representative curves for the concentration dependence of the inhibition of GIVA cPLA_2_ by 2-oxoesters **17a**, **17b** and **17d** were fit to sigmoidal curves and are presented in Fig. 6.

## Discussion

Methyl 2-oxopalmitate **10e** (entry 1, Table 1) weakly inhibited, at a high concentration, both the intracellular enzymes GIVA cPLA_2_ and GVIA iPLA_2_. However, 2-oxopalmitic acid **12e** (entry 2, Table 1) inhibited weakly, but selectively, GIVA cPLA_2_. Interestingly, when the 2-oxoester functionality was combined with a long aliphatic chain together with a free carboxyl group at a distance of three carbon atoms, potent inhibition of GIVA cPLA_2_ was observed and the inhibitor **17a** (GK161) showed a *X*_I_(50) value of 0.00008 (entry 3, Table 1). In addition, this inhibitor was selective and did not inhibit the activities of GVIA iPLA_2_ and the secreted GV sPLA_2_. This selectivity is in agreement with our previous observations that 2-oxoamides containing a free carboxyl group selectively inhibit GIVA cPLA_2_^28, 30^. Given that for the most potent 2-oxoamides present *X*_I_(50) values are not lower than 0.003^30^, the present 2-oxoester was proven to be a much more potent inhibitor of GIVA cPLA_2_. The corresponding 2-hydroxy ester derivative **20** did not present any inhibition of either GIVA cPLA_2_ or GVIA iPLA_2_ (entry 4, Table 1), demonstrating the importance of the oxoester functionality for the inhibition.

When the long aliphatic chain was replaced by a chain of a similar size containing an aromatic ring, the inhibitory activity over GIVA cPLA_2_ was considerably reduced (entry 5, Table 1). Compounds **16c** and **19** containing a medium chain carrying an aromatic ring and a protected carboxyl group (either ethyl ester or *tert*-butyl ester) totally abolished any inhibitory activity (entries 6 and 7, Table 1). In accord with our expectation, the replacement of the long aliphatic chain by a more drug-like chain of four carbon atoms carrying a biphenyl system led again to a potent and selective inhibition of GIVA cPLA_2_ (entry 8, Table 1). Inhibitor **17d** (GK200) was found to be eight times less potent than **17a** showing a *X*_I_(50) value of 0.00068. To extend the structure-activity relationship studies, we either increased the distance between the free carboxyl group and the oxoester functionality or decreased the distance between the aromatic rings and the oxoester functionality. Inhibitor **17e** (GK433) proved to be highly potent, slightly better than **17a**, presenting a *X*_I_(50) value of 0.00007 (entry 9, Table 1). The importance of the four-carbon atoms distance between the free carboxyl group and the oxoester functionality was clearly demonstrated by the inhibitor **17f** (GK452), which presented highly potent inhibition of GIVA cPLA_2_ with a *X*_I_(50) value of 0.000078 (entry 10, Table 1). Decrease of the distance between the biphenyl aromatic system and the oxoester functionality (compounds **17g** and **17h**) resulted in considerable reduction of the potency (entries 11 and 12, Table 1). All the highly potent GIVA cPLA_2_ inhibitors **17a**, **17d**, **17e** and **17f** presented selectivity, because none of them exhibited any appreciable inhibition of GVIA iPLA_2_. In addition, none of the synthesized and tested 2-oxoesters inhibited GV sPLA_2_.

Since both the intracellular enzymes GIVA cPLA_2_ and GVIA iPLA_2_ are serine hydrolases and both utilize a catalytic dyad in their catalytic mechanism, it is likely that cross reactivity may be observed for inhibitors designed to carry a functionality targeting the active site serine. Indeed, such cross reactivity has been observed for several inhibitors containing an activated carbonyl group initially developed to target GIVA cPLA_2_. For example, arachidonoyl trifluoromethyl ketone was found to inhibit not only GIVA cPLA_2_, but also GVIA iPLA_2_. It is apparent that the presence of other groups able to develop appropriate hydrophobic and/or hydrophilic interactions contributes to the overall binding of the inhibitor to the enzyme, determining the inhibitory selectivity over GIVA cPLA_2_ or GVIA iPLA_2_. We have previously shown that pentafluoroethyl or trifluoromethyl ketones of a four-carbon atom chain carrying an aromatic ring are selective inhibitors of GVIA iPLA_2_^46–48^. Inspired by the structures of FKGK11^46^ and FKGK18^47^, we designed simple methyl 2-oxoesters with a linker of four methylene groups between the activated carbonyl group and the aromatic ring. Unfortunately, compound **10a** (entry 13, Table 1) carrying a phenyl ring only weakly inhibited GVIA iPLA_2_ at a high concentration. On the contrary, compound **10b** (GK451) (entry 14, Table 1) carrying a naphthalene ring presented interesting inhibition of GVIA iPLA_2_ with a *X*_I_(50) value of 0.0052. At the same time, it presents selectivity, because it only weakly inhibits GIVA cPLA_2_ at a high concentration (55% at 0.091 mole fraction), while it does not inhibit at all GV sPLA_2_.

**Figure 7:**
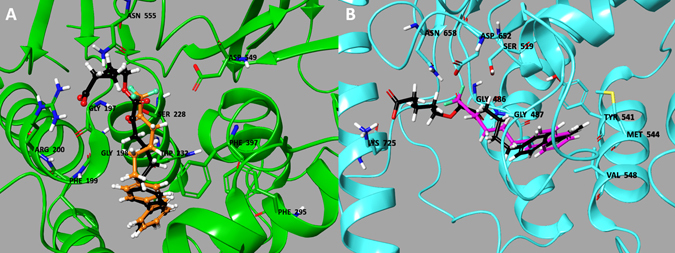
Binding mode of inhibitor **17f** in the active site of (**a**) GIVA cPLA_2_ and (**b**) GVIA iPLA_2_.

To better understand the interaction of 2-oxoesters with GIVA cPLA_2_ and GVIA iPLA_2_, the most potent GIVA cPLA_2_ inhibitor **17f** was docked in the active site of either GIVA cPLA_2_ or GVIA iPLA_2_. For the docking calculations, the structures of GIVA cPLA_2_ and GVIA iPLA_2_ with two different fluoroketone compounds in the active site were used (GK174: orange color in Fig. 7a and FKGK18: magenta color in Fig. 7b). The binding mode of these two fluoroketones was validated using H/D exchange and MD simulations in a previously published study^49^. A theoretical score of 10.2 kcal/mol indicated that **17f** is a tight binder for GIVA cPLA_2_. The oxoester moiety forms hydrogen-bonding with the oxyanion hole (Gly197/Gly198), while the carboxylic moiety interacts with Arg200, which was found to stabilize the phosphate group of a phospholipid substrate molecule^39^. Compared to GK174 (orange color in Fig. 7a) the addition of the carboxylic moiety is responsible for increasing the potency of **17f** by 10-fold. This compound exhibits no activity towards GVIA iPLA_2_ and it received a low theoretical binding score of 6.3 kcal/mol indicating that is a weak binder. Compared to fluoroketone FKGK18 (magenta color in Fig. 7b) the addition of the carboxylic moiety increases the size of the compound and it cannot be accommodated in the active site of GVIA iPLA_2_.

All the above data, clearly demonstrate that 2-oxoesters consisting of a quite long chain (aliphatic or incorporating aromatic systems like the biphenyl system) in combination with a free carboxyl group at a distance of four or three carbon-atoms from the oxoester functionality are highly potent and selective inhibitors of GIVA cPLA_2_. Decreasing the size of the synthetic compound and eliminating the free carboxyl group may change the selectivity. Indeed, a methyl 2-oxoester based on a short chain carrying a naphthalene ring was found to inhibit preferentially GVIA iPLA_2_. In other words, it seems that the selectivity of compounds based on the 2-oxoester functionality may be tuned choosing the structural features that ensure the appropriate interactions with each enzyme (either GIVA cPLA_2_ or GVIA iPLA_2_).

To compare our novel highly potent 2-oxoester inhibitors of GIVA cPLA_2_ with the existing inhibitors, we studied the benchmark GIVA cPLA_2_ inhibitor **4** in our mixed-micelle assay. This inhibitor, developed by Lehr^32^, is the most potent inhibitor in the literature presenting an IC_50_ value of 4.3 nM in a vesicle assay^32^. In the mixed micelle assay, it was proved equipotent with oxoester **17a** with a *X*_I_(50) value of 0.00008 (entry 15, Table 1). In addition, several 2-oxoesters were found to be more potent than the other benchmark GIVA cPLA_2_ inhibitor **1** (pyrrophenone), which presents an *X*_I_(50) value of 0.002^26, 27^. Another important property of a GIVA cPLA_2_ inhibitor, is the ClogP value, which is a measure of the hydrophobicity. ClogP represents the calculated partition coefficient in octanol/water on a logarithmic scale. Usually, GIVA cPLA_2_ inhibitors suffer from high lipophilicity. For example, the ClogP value of inhibitor **4** is 8.50, while pyrrophenone **1** and giripladib **2c** present high lipophilicities too (ClogP 8.29 and 10.75, respectively). Inhibitors with such high values are not expected to present favorable ADME properties according to Lipinski’s rule of five^50^. Although 2-oxoesters **17a** and **17e** contain a long aliphatic chain, they present lower lipophilicity (ClogP 6.76 and 6.68, respectively), while the 2-oxoesters **17d** and **17f** carrying the biphenyl system have considerably lower ClogP values (4.78 and 4.70, respectively). The logP value of **17f**, measured by HPLC, was found 3.5. Thus, the lipophilicity of **17f** is encouraging and this inhibitor is the first example of a highly potent GIVA cPLA_2_ inhibitor, which presents a ClogP value lower than 5.

**Figure 8:**
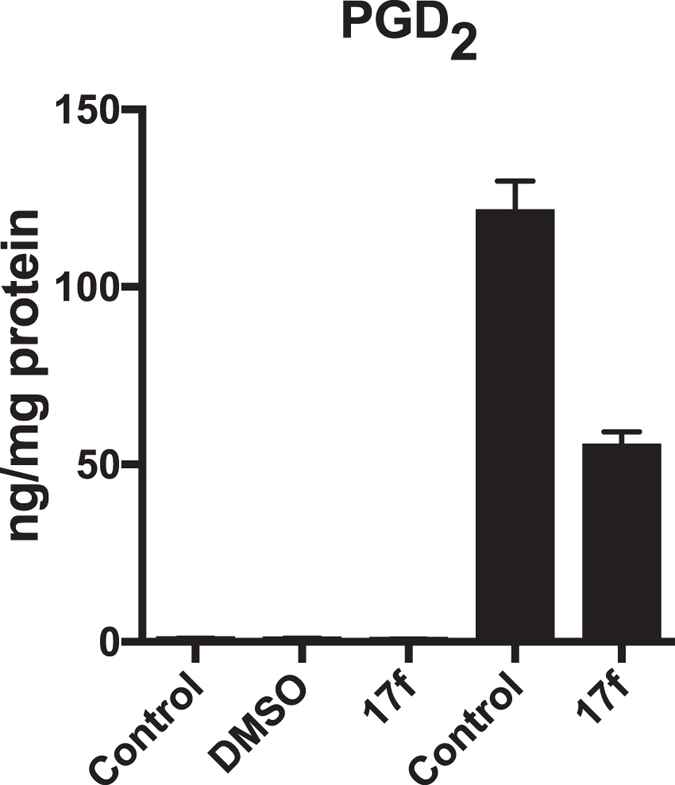
Inhibitor **17f** inhibits KLA-elicited prostaglandin D_2_ biosynthesis by macrophages. Macrophages were pre-treated with media (control), vehicle control (DMSO, 0.1%) or inhibitor **17f** (5 μM) 1 hr before KLA (100 ng/mL, ■) or mock (□) treatment. Supernatants were collected 24 hr following stimulation for eicosanoid quantification. Graph displays the mean ± SEM of a single experiment containing technical duplicates that is representative of 2 independent experiments. * indicates statistical difference compared to KLA treatment (P ≤ 0.05).

The cellular effect of the most potent GIVA cPLA_2_ inhibitor **17f** on eicosanoid biosynthesis was studied in macrophages. RAW264.7 macrophages were used as a model system to determine if **17f** displays inhibitory activity toward GIVA cPLA_2_ *in vivo*. It is well established that the toll-like receptor 4 (TLR4)-specific agonist Kdo2-lipid A (KLA) leads to GIVA cPLA_2_ activation^51, 52^ and release of arachidonic acid in macrophages that is then converted into eicosanoids by cyclooxygenase-2^53–55^. Previous work has demonstrated that the major eicosanoid produced by KLA stimulated RAW264.7 macrophages is prostaglandin D_2_ (PGD_2_)^56^. The high levels of PGD_2_ compared to background in culture supernatants following KLA stimulation makes it an ideal marker for GIVA cPLA_2_ activity in macrophages. Inhibitor **17f** did not show cellular toxicity at any concentrations tested as measured by trypan blue exclusion (data not shown). RAW264.7 macrophages were pre-treated with vehicle control, DMSO or **17f** (5 μM) for one hour prior to stimulation with KLA (100 ng/mL). Culture supernatants were collected after 24 hours for eicosanoid quantification by LC-MS/MS. Treatment with inhibitor **17f** resulted in over 50% decrease in KLA-elicited PGD_2_ production by macrophages (Fig. 8). A similar reduction in other minor products including PGE_2_, PGF_2α_, 11-HETE and 15-HETE was observed (data not shown), suggesting that the inhibition was not specific to PGD_2_. This data is consistent with **17f** inhibition of GIVA cPLA_2_ in living cells.

In conclusion, we describe a novel class of GIVA cPLA_2_ inhibitors based on the 2-oxoester functionality. This reactive functionality in combination with a long aliphatic chain or a chain carrying an appropriate aromatic system, such as the biphenyl system, and a free carboxyl group leads to highly potent and selective GIVA cPLA_2_ inhibitors. Inhibitors **17a**, **17e** and **17f** present *X*_I_(50) values of 0.00007–0.00008 and are equipotent to the most potent known GIVA cPLA_2_ inhibitor. In particular, inhibitors incorporating the biphenyl system, like **17f**, present interesting favorable lipophilicity (ClogP values lower than 5). The novel highly potent and selective GIVA cPLA_2_ inhibitors may be excellent tools for the study of the role of the enzyme in cells and in animals and may contribute to the development of novel medicinal agents for the treatment of inflammatory diseases.

## Methods

### General

Chromatographic purification of products was accomplished using Merck Silica Gel 60 (70–230 or 230–400 mesh). Thin-layer chromatography (TLC) was performed on Silica Gel 60 F254 aluminum plates. TLC pots were visualized with UV light and/or phosphomolybdic acid in EtOH. Melting points were determined using a Büchi 530 apparatus and were uncorrected. ^1^H and ^13^C NMR spectra were recorded on a Varian Mercury (200 MHz and 50 MHz respectively) in CDCl_3_. Chemical shifts are given in ppm, and coupling constants (*J*) in Hz. Peak multiplicities are described as follows: s, singlet, d, doublet, t, triplet and m, multiplet. Electron spray ionization (ESI) mass spectra were recorded on a Finnigan, Surveyor MSQ Plus spectrometer. Dichloromethane was dried by standard procedures and stored over molecular sieves. All other solvents and chemicals were reagent grade and used without further purification. The purity of all compounds subjected to biological tests was determined by analytical HPLC, and was found to be ≥95%. HPLC analyses were carried out on a Shimadzu LC-2010AHT system and a Merck Chromolith Performance (100 × 4.6 mm) analytical column, using H_2_O/MeOH 10/90 v/v, at a flow rate of 1.0 mL/min. HRMS spectra were recorded on a Bruker Maxis Impact QTOF Spectrometer.

Compounds **8a** ^57^, **8b** ^57^, **9e** ^58^, **11a** ^57^, **11b** ^57^, **13a** ^59^, **13b** ^60^ have been described elsewhere and their analytical data are in accordance with literature.

### Synthesis of cyanohydrins 8c,d

To a stirred solution of aldehyde **7c,d** (1.0 mmol) in CH_2_Cl_2_ (1.4 mL), an aqueous solution of NaHSO_3_ (0.25 mL, 1.5 mmol) was added and the mixture was stirred for 30 min at room temperature. The organic solvent was evaporated under reduced pressure and H_2_O (1 mL) was added. The mixture was cooled to 0 °C and an aqueous solution of KCN (0.25 mL, 1.5 mmol) was added within 2 h under vigorous stirring. The reaction was stirred for 18 h at room temperature and then, water (10 mL) was added and extracted with CH_2_Cl_2_ (3 × 10 mL). The combined organic phases was washed with brine (30 mL), dried over Na_2_SO_4_ and evaporated under reduced pressure. The residue was purified by flash column chromatography [ethyl acetate (EtOAc)/petroleum ether (bp 40–60 °C), 2:8].

### 6-([1,1′-Biphenyl]-4-yl)-2-hydroxyhexanenitrile (8c)

Yield 80%; White solid; mp: 85–87 °C; ^1^H NMR (200 MHz, CDCl_3_): δ 7.67–7.14 (m, 9 H), 4.45 (t, J = 6.9 Hz, 1 H), 3.90 (br s, 1 H), 2.69 (t, J = 7.1 Hz, 2 H), 1.89 (q, J = 7.2 Hz, 2 H), 1.79–1.39 (m, 4 H); ^13^C NMR (50 MHz, CDCl_3_): δ 141.0, 140.9, 138.8, 129.0, 128.7, 127.0, 126.9, 126.5, 119.9, 61.1, 35.2, 34.9, 30.6, 24.2; MS (m/z, ESI): [M + NH_4_]^+^ calcd. for C_18_H_19_NO, 283.2; found, 283.3; analysis (calcd., found for C_18_H_19_NO): C (81.47, 81.18), H (7.22, 7.41), N (5.28, 5.33).

### 5-([1,1′-Biphenyl]-4-yl)-2-hydroxypentanenitrile (8d)

Yield 76%; White solid; mp: 80–82 °C; ^1^H NMR (200 MHz, CDCl_3_): δ 7.64–7.23 (m, 9 H), 4.48 (t, J = 6.9 Hz, 1 H), 3.80 (br s, 1 H), 2.64 (t, J = 7.0 Hz, 2 H), 1.84 (q, J = 7.0 Hz, 2 H), 1.69–1.57 (m, 2 H); ^13^C NMR (50 MHz, CDCl_3_): δ 141.3, 140.8, 138.4, 129.1, 128.7, 127.6, 127.2, 126.8, 118.5, 61.9, 35.1, 34.5, 23.8; MS (m/z, ESI): [M + NH_4_]^+^ calcd. for C_17_H_17_NO 269.2; found, 269.2; analysis (calcd., found for C_17_H_17_NO): C (81.24, 81.02), H (6.82, 6.99), N (5.57, 5.69).

### Synthesis of 2-hydroxy esters 9a-d

Cyanohydrin **8a-d** (1 mmol) was dissolved in methanolic solution of HCl (10 mL, 4 N) and the reaction mixture was stirred for 24 h at room temperature. The organic solvent was evaporated *in vacuo* and the remaining solid was dissolved in diethyl ether (10 mL) and re-evaporated. Dilution and evaporation was repeated twice. Then, the product was purified by flash column chromatography [EtOAc-petroleum ether (bp 40–60 °C), 2:8].

### Methyl 2-hydroxy-6-phenylhexanoate (9a)

Yield 61%; Yellow oil; ^1^H NMR (200 MHz, CDCl_3_): δ 7.37–7.04 (m, 5 H), 4.23–4.10 (m, 1 H), 3.77 (s, 3 H), 2.74 (br s, 1 H), 2.62 (t, J = 7.1 Hz, 2 H), 1.92–1.25 (m, 6 H); ^13^C NMR (50 MHz, CDCl_3_): δ 175.7, 142.3, 128.3, 128.2, 125.6, 70.3, 52.4, 35.7, 34.1, 31.1, 24.4; MS (m/,z ESI): [M + NH_4_]^+^ calcd. for C_13_H_18_O_3_ 240.2 found, 240.2; analysis (calcd., found for C_13_H_18_O_3_): C (70.24, 70.01), H (8.16, 8.29).

### Methyl 2-hydroxy-6-(naphthalen-2-yl)hexanoate (9b)

Yield 73%; Colorless oil; ^1^H NMR (200 MHz, CDCl_3_): δ 7.90–7.20 (m, 7 H), 4.30–4.02 (m, 1 H), 3.76 (s, 3 H), 3.35 (br s, 1 H), 2.97–2.75 (m, 2 H), 1.97–1.34 (m, 6 H); ^13^C NMR (50 MHz, CDCl_3_): δ 175.6, 139.8, 133.5, 127.7, 127.5, 127.3, 127.2, 126.2, 125.8, 125.0, 70.3, 52.4, 35.8, 34.1, 30.9, 24.4; MS (m/z, ESI): [M + Na]^+^ calcd. for C_17_H_20_O_3_ 295.1, found, 295.2; analysis (calcd., found for C_17_H_20_O_3_): C (74.97, 74.72), H (7.40, 7.62).

### Methyl 6-([1,1′-biphenyl]-4-yl)-2-hydroxyhexanoate (9c)

Yield 69%; Colorless oil; ^1^H NMR (200 MHz, CDCl_3_): δ 7.70–7.06 (m, 9 H), 4.45 (t, J = 7.0 Hz, 1 H), 3.79 (s, 3 H), 3.00 (br s, 1 H), 2.69 (t, J = 7.1 Hz, 2 H), 1.89 (q, J = 7.5 Hz, 2 H), 1.79–1.36 (m, 4 H); ^13^C NMR (50 MHz, CDCl_3_): δ 175.7, 141.0, 140.9, 138.7, 129.0, 128.7, 127.0, 126.9, 70.4, 52.5, 35.2, 34.9, 30.7, 24.2; MS (m/z, ESI): [M + Na]^+^ calcd. for C_19_H_22_O_3_ 321.1, found, 321.2; analysis (calcd., found for C_19_H_22_O_3_): C (80.82, 80.61), H (7.85, 7.98).

### Methyl 5-([1,1′-biphenyl]-4-yl)-2-hydroxypentanoate (9d)

Yield 71%; Colorless oil; ^1^H NMR (200 MHz, CDCl_3_): δ 7.69–7.18 (m, 9 H), 4.40 (t, J = 6.9 Hz, 1 H), 3.76 (s, 3 H), 3.54 (brs, 1 H), 2.65 (t, J = 7.1 Hz, 2 H), 1.84 (q, J = 7.1 Hz, 2 H), 1.64–1.36 (m, 2 H); ^13^C NMR (50 MHz, CDCl_3_): δ 176.1, 141.3, 140.8, 138.2, 129.0, 128.9, 127.5, 127.0, 126.8, 70.3, 52.3, 35.5, 34.9, 24.4; MS (m/z, ESI): [M + Na]^+^ calcd. for C_18_H_20_O_3_ 307.1, found, 307.2; analysis (calcd., found for C_18_H_20_O_3_): C (76.03, 75.83), H (7.09, 7.27).

### Synthesis of 2-oxoesters 10α, 10b, 10e, 16a-h, 19

To a stirred solution of 2-hydroxy esters **9a, 9b**, **9e**, **15a-h**, **18** (1 mmol) in dry CH_2_Cl_2_ (10 mL) was added Dess-Martin periodinane (1.1 mmol, 0.47 g) and the reaction mixture was stirred for 1.5 h at room temperature. Then, CH_2_Cl_2_ (5 mL) was added and the organic phase was washed with a mixture of Na_2_S_2_O_3_ 10% and NaHCO_3_ 10% (15 mL, 1:1, v/v). Τhe aqueous phase was washed with CH_2_Cl_2_ (15 mL) and all the organic phases were collected, dried (Na_2_SO_4_) and evaporated under reduced pressure. The residue was purified by flash column chromatography [EtOAc-petroleum ether (bp 40–60 °C), 2:8].

### Methyl 2-oxo-6-phenylhexanoate (10a, GK437)

Yield 66%; Colorless oil; ^1^H NMR (200 MHz, CDCl_3_): δ 7.40–7.08 (m, 5 H), 3.84 (s, 3 H), 2.85 (t, J = 6.4 Hz, 2 H), 2.62 (t, J = 6.5 Hz, 2 H), 1.78–1.58 (m, 4 H); ^13^C NMR (50 MHz, CDCl_3_): δ 194.0, 161.4, 141.8, 128.3, 128.1, 125.8, 52.9, 39.1, 35.5, 30.6, 22.5; MS (m/z, ESI): [M + NH_4_]^+^ calcd. for C_13_H_16_O_3_ 238.1, found, 238.2; HRMS (m/z, ESI): [M + Na]^+^ calcd. for C_13_H_16_O_3_, 243.0992; found, 243.0994; analysis (calcd., found for C_13_H_16_O_3_): C (70.89, 70.58), H (7.32, 7.46).

### Methyl 6-(naphthalen-2-yl)-2-oxohexanoate (10b, GK451)

Yield 73%; Colorless oil; ^1^H NMR (200 MHz, CDCl_3_): δ 7.90–7.10 (m, 7 H), 3.85 (s, 3 H), 2.92–2.71 (m, 4 H), 1.83–1.49 (m, 4 H); ^13^C NMR (50 MHz, CDCl_3_): δ 194.0, 161.4, 139.2, 133.5, 131.9, 127.9, 127.5, 127.4, 127.2, 126.3, 125.9, 125.1, 52.9, 39.1, 35.6, 30.4, 22.5; MS (m/z, ESI): [(M + NH_4_)^+^] calcd. for C_17_H_18_O_3_ 288.2, found, 288.2; HRMS (m/z, ESI): [M + Na]^+^ calcd. for C_17_H_18_O_3_, 293.1148; found, m/z 293.1149; analysis (calcd., found for C_17_H_18_O_3_): C (75.53, 75.32), H (6.71, 6.95).

### Methyl 2-oxohexadecanoate (10e)

Yield 73%; White solid; mp: 53–55 °C; ^1^H NMR (200 MHz, CDCl_3_): δ 3.85 (s, 3 H), 2.82 (t, J = 7.2 Hz, 2 H), 1.70–1.51 (m, 2 H), 1.37–1.16 (m, 22 H), 0.86 (t, J = 7.0 Hz, 3 H); ^13^C NMR (50 MHz, CDCl_3_): δ 194.3, 161.5, 52.8, 39.3, 31.9, 29.6, 29.5, 29.4, 29.3, 29.2, 28.9, 22.9, 22.6, 14.1; MS (m/z, ESI): [M + NH_4_]^+^ calcd. for C_17_H_32_O_3_ 302.3; found, 302.3^61^.

### 4-(*tert*-Butoxy)-4-oxobutyl 2-oxohexadecanoate (16a)

Yield 87%; Colorless oil, ^1^H NMR (200 MHz, CDCl_3_): δ 4.27 (t, J = 6.0 Hz, 2 H), 2.81 (t, J = 7.8 Hz, 2 H), 2.33 (t, J = 6.0 Hz, 2 H), 2.04 (quint, J = 6.0 Hz, 2 H), 1.70–1.50 (m, 2 H), 1.44 (s, 9 H), 1.40–1.15 (m, 22 H), 0.86 (t, J = 7.0 Hz, 3 H); ^13^C NMR (50 MHz, CDCl_3_): δ 194.5, 171.8, 161.2, 80.6, 65.2, 39.3, 31.9, 31.6, 29.6, 29.5, 29.4, 29.3, 29.2, 28.9, 28.0, 23.8, 22.9, 22.6, 14.1; MS (m/z, ESI): [M + NH_4_]^+^ calcd. for C_24_H_44_O_5_ 430.4; found, 430.4; analysis (calcd., found for C_24_H_44_O_5_): C (69.86, 69.6), H (10.75, 10.92).

Compound **16b** was not isolated and used directly in the next step.

### 4-(*tert*-Butoxy)-4-oxobutyl 2-oxo-6-phenylhexanoate (16c, GK192)

Yield 77%; Yellow oil; ^1^H NMR (200 MHz, CDCl_3_): δ 7.35–7.10 (m, 5 H), 4.26 (t, J = 8.0 Hz, 2 H), 2.84 (t, J = 6.0 Hz, 2 H), 2.70–2.55 (m, 2 H), 2.32 (t, J = 6.0 Hz, 2 H), 2.00 (quint, J = 6.0 Hz, 2 H), 1.70–1.60 (m, 4 H), 1.44 (s, 9 H), ^13^C NMR (50 MHz, CDCl_3_): δ 194.1, 171.7, 161.1, 141.8, 128.3, 128.2, 125.8, 80.6, 65.3, 39.1, 35.5, 31.6, 30.6, 28.0, 23.8, 22.5; HRMS (m/z, ESI): [M + Na]^+^calcd. for C_20_H_28_O_5_ 371.1829; found, 371.1831; analysis (calcd., found for C_20_H_28_O_5_): C (68.94, 68.66), H (8.10, 8.29).

### 4-*tert*-Butoxy-4-oxobutyl 6-(biphenyl-4-yl)-2-oxohexanoate (16d)

Yield 86%; Colorless oil; ^1^H NMR (200 MHz, CDCl_3_): δ 7.96–7.16 (m, 9 H), 4.28 (t, J = 6.4 Hz, 2 H), 2.97–2.83 (m, 2 H), 2.77–2.62 (m, 2 H), 2.42–2.28 (m, 2 H), 2.11–1.55 (m, 6 H), 1.45 (s, 9 H); ^13^C NMR (50 MHz, CDCl_3_): δ 194.1, 171.8, 161.0, 140.9, 138.7, 129.0, 128.9, 128.7, 128.6, 127.2, 127.0, 126.9, 80.6, 65.3, 39.1, 35.1, 31.6, 30.5, 28.0, 23.7, 22.5; MS (m/z, ESI): [M + Na]^+^ calcd. for C_26_H_32_O_5_ 447.2; found, 447.0; analysis (calcd., found for C_26_H_32_O_5_): C (73.56, 73.35), H (7.60, 7.78).

### 5-(*tert*-Butoxy)-5-oxopentyl 2-oxohexadecanoate (16e)

Yield 78%; White oil; ^1^H NMR (200 MHz, CDCl_3_): δ 4.23 (t, J = 6.9 Hz, 2 H), 2.79 (t, J = 7.3 Hz, 2 H), 2.24 (t, J = 6.9 Hz, 2 H), 1.80–1.45 (m, 4 H), 1.44 (s, 9 H), 1.30–1.15 (s, 24 H), 0.85 (t, J = 7.0 Hz, 3 H); ^13^C NMR (50 MHz, CDCl_3_): δ 194.8, 172.6, 161.5, 80.5, 66.0, 39.5, 35.0, 32.1, 29.9, 29.8, 29.6, 29.5, 29.1, 28.3, 27.9, 23.1, 22.9, 21.6, 14.3; MS (m/z, ESI): [M + NH_4_]^+^ calcd. for C_25_H_46_O_5_ 444.4; found, 444.3; analysis (calcd., found for C_25_H_46_O_5_): C (70.38, 70.17), H (10.87, 11.05).

### 5-(*tert*-Butoxy)-5-oxopentyl 6-([1,1′-biphenyl]-4-yl)-2-oxohexanoate (16f)

Yield 63%; Colorless oil; ^1^H NMR (200 MHz, CDCl_3_): δ 7.66–7.19 (m, 9 H), 4.25 (t, J = 6.0 Hz, 2 H), 2.87 (t, J = 6.3 Hz, 2 H), 2.68 (t, J = 6.1 Hz, 2 H), 2.26 (t, J = 7.0 Hz, 2 H), 1.86–1.53 (m, 8 H), 1.44 (s, 9 H); ^13^C NMR (50 MHz, CDCl_3_): δ 194.3, 172.4, 161.1, 141.0, 138.8, 129.0, 128.8, 128.7, 127.2, 127.0, 126.9, 80.3, 65.9, 39.1, 35.2, 34.8, 30.6, 28.1, 27.7, 22.5, 21.3; MS (m/z, ESI): [M + NH_4_]^+^ calcd. for C_27_H_34_O_5_ 456.3; found, 456.3; analysis (calcd., found for C_27_H_34_O_5_): C (73.95, 73.75), H (7.81, 7.99).

### 4-(*tert*-Butoxy)-4-oxobutyl 5-([1,1′-biphenyl]-4-yl)-2-oxopentanoate (16 g)

Yield 65%; Colorless oil; ^1^H NMR (200 MHz, CDCl_3_): δ 7.64–7.20 (m, 9 H), 4.27 (t, J = 6.4 Hz, 2 H), 2.88 (t, J = 7.2 Hz, 2 H), 2.70 (t, J = 7.5 Hz, 2 H), 2.33 (t, J = 7.2 Hz, 2 H), 2.10–1.90 (m, 4 H), 1.44 (s, 9 H); ^13^C NMR (50 MHz, CDCl_3_): δ 194.3, 172.1, 161.3, 141.2, 140.5, 139.3, 129.1, 128.9, 127.4, 127.3, 127.2, 80.9, 65.6, 38.8, 34.6, 31.9, 28.3, 24.7, 24.0; MS (m/z, ESI): [M + NH_4_]^+^ calcd. for C_25_H_30_O_5_ 428.2; found, 428.3; analysis (calcd., found for C_25_H_30_O_5_): C (73.15, 72.97), H (7.37, 7.56).

### 5-(*tert*-Butoxy)-5-oxopentyl 5-([1,1′-biphenyl]-4-yl)-2-oxopentanoate (16 h)

Yield 61%; Colorless oil; ^1^H NMR (200 MHz, CDCl_3_): δ 7.64–7.20 (m, 9 H), 4.24 (t, J = 6.0 Hz, 2 H), 2.87 (t, J = 6.3 Hz, 2 H), 2.69 (t, J = 7.5 Hz, 2 H), 2.25 (t, J = 5.9 Hz, 2 H), 2.20–1.90 (m, 2 H), 1.90–1.54 (m, 4 H), 1.44 (s, 9 H); ^13^C NMR (50 MHz, CDCl_3_); δ 194.4, 172.7, 161.3, 141.2, 140.5, 139.3, 129.1, 128.9, 127.4, 127.3, 127.2, 80.6, 66.2, 38.8, 35.1, 34.6, 28.3, 27.9, 24.7, 21.6; MS (m/z, ESI): [M + NH_4_]^+^ calcd. for C_26_H_32_O_5_ 442.3; found, 442.3; analysis (calcd., found for C_26_H_32_O_5_): C (73.56, 73.32), H (7.60, 7.82).

### 4-Ethoxy-4-oxobutyl 2-oxo-6-phenylhexanoate (19, GK194)

Yield 73%; Yellowish oil, ^1^H NMR (200 MHz, CDCl_3_): δ 7.30–7.10 (m, 5 H), 4.28 (t, J = 8.0 Hz, 2 H), 4.13 (q, J = 6.0 Hz, 2 H) 2.84 (t, J = 6.0 Hz, 2 H), 2.70–2.55 (m, 2 H), 2.41 (t, J = 8.0 Hz, 2 H), 2.15–1.95 (m, 2 H), 1.70–1.55 (m, 4 H), 1.24 (t, J = 6.0 Hz, 3 H); ^13^C NMR (50 MHz, CDCl_3_): δ 194.0, 172.4, 161.0, 141.8, 128.3, 128.2, 125.7, 65.2, 60.5, 39.0, 35.5, 30.6, 30.5, 23.6, 22.5, 14.1; MS (m/z, ESI): [M + NH_4_]^+^calcd. for C_18_H_24_O_5_ 338.2; found, 338.2;HRMS (m/z, ESI): [M + Na]^+^ calcd for C_18_H_24_O_5_ 343.1516; found, 343.1512; analysis (calcd., found for C_18_H_24_O_5_): C (67.48, 67.19), H (7.55, 7.61).

### Synthesis of 2-hydroxy acids 11α-e

To a stirred solution of 2-hydroxy ester **9a-e** (1 mmol) in methanol (10 mL), aqueous NaOH (1.1 mL, 1 N) was added and the reaction mixture was stirred overnight at room temperature. The organic solvent was evaporated *in vacuo* to dryness and then aqueous HCl 1 N was added until acidic pH. The aqueous phase was washed with EtOAc (3 × 10 mL). Finally, the organic phase was dried (Na_2_SO_4_) and evaporated under reduced pressure.

### 6-([1,1′-Biphenyl]-4-yl)-2-hydroxyhexanoic acid (11c)

Yield 99%; White solid; mp: 143–145 °C; ^1^H NMR (200 MHz, CDCl_3_): δ 10.43 (s, 1 H), 7.67–7.02 (m, 9 H), 5.00 (br s, 1 H) 4.22 (t, J = 6.0 Hz, 1 H), 2.59 (t, J = 7.2 Hz, 2 H), 2.03–1.37 (m, 6 H); ^13^C NMR (50 MHz, CDCl_3_): δ 176.6, 141.2, 140.6, 138.1, 129.1, 128.3, 128.2, 126.5, 126.4, 69.8, 34.9, 33.6, 30.8, 24.3; MS (m/z, ESI): [M-H]^−^ calcd. for C_18_H_20_O_3_ 283.1; found, 283.1; analysis (calcd., found for C_18_H_20_O_3_): C (76.03, 75.85), H (7.09, 7.25).

### 5-([1,1′-Biphenyl]-4-yl)-2-hydroxypentanoic acid (11d)

Yield 64% (over two steps); Light violet viscous oil; ^1^H NMR (200 MHz, CDCl_3_): δ 10.60 (s, 1 H), 7.66–7.06 (m, 9 H), 4.98 (s, 1 H), 4.29 (t, J = 6.0 Hz, 1 H), 2.68 (t, J = 6.4 Hz, 2 H), 1.99–1.68 (m, 4 H); ^13^C NMR (50 MHz, CDCl_3_): δ 178.4, 141.3, 141.2, 139.0, 129.1, 129.0, 128.9, 127.3, 127.2, 70.3, 35.3, 33.9, 26.8; MS (m/z, ESI): [M-H]^−^ calcd. for C_17_H_18_O_3_ 269.1; found, 269.1; analysis (calcd., found for C_17_H_18_O_3_): C (75.53, 75.31), H (6.71, 6.87).

### 2-Oxohexadecanoic acid (12e)

To a stirred solution of **9e** (0.35 mmol, 100 mg) in MeOH (3.5 mL), aqueous Cs_2_CO_3_ 20% (w/v) (1.7 mL, 1.0 mmol) was added, and the reaction mixture was stirred at room temperature. The reaction progress was monitored by TLC, until completion. The organic solvent was evaporated *in vacuo* to dryness, water was added (10 mL) and then aqueous HCl 1 N was added until acidic pH. The aqueous phase was washed with EtOAc (3 × 10 mL). Finally, the organic phase was dried over Na_2_SO_4_ and evaporated under reduced pressure. Yield 32%; White solid; mp: 66–68 °C; ^1^H NMR (200 MHz, CDCl_3_): δ 9.02 (br s, 1 H), 2.93 (t, J = 7.2 Hz, 2 H), 1.76–1.51 (m, 2 H), 1.43–1.05 (m, 22 H), 0.88 (t, J = 6.6 Hz, 3 H); ^13^C NMR (50 MHz, CDCl_3_): δ 194.8, 162.5, 39.3, 31.9, 29.6, 29.5, 29.4, 29.3, 29.2, 28.9, 22.9, 22.7, 14.1; MS (m/z, ESI): [M-H]^−^ calcd. for C_16_H_30_O_3_ 269.2; found, 269.2^62^.

### Synthesis of 2-hydroxy esters 15a-h and 18

To a stirred solution of 2-hydroxy acids **11a, 11c, 11d, 13a,b** (1 mmol) in tetrahydrofuran (THF) (6 mL), water (0.6 mL) and few drops of aqueous CsCO_3_ 20% (w/v) were added in order to adjust pH in neutral value. The organic solvent was evaporated *in vacuo* and the residue was dissolved in N,N-dimethylformamide (DMF) (15 mL). Subsequently, *tert*-butyl 5-bromoalkanooate **14a,b** or ethyl 4-bromobutyrate (1.2 mmol) was added and the reaction mixture was refluxed for 72 h. Water (20 mL) was then added and the reaction mixture was washed with EtOAc (2 × 20 mL). The organic phase was dried (Na_2_SO_4_) and evaporated under reduced pressure. The residue was purified by flash column chromatography [EtOAc-petroleum ether (bp 40–60 °C), 1:9 or 2:8].

### 4-(*tert*-Butoxy)-4-oxobutyl 2-hydroxyhexadecanoate (15a)

Yield 44%; Yellow oil, ^1^H NMR (200 MHz, CDCl_3_): δ 4.25–4.10 (m, 3 H), 2.73 (br s, 1 H), 2.30 (t, J = 6.0 Hz, 2 H), 1.94 (qu, J = 6.0 Hz, 2 H), 1.60–1.45 (m, 2 H), 1.43 (s, 9 H), 1.40–1.20 (m, 24 H), 0.86 (t, J = 6.0 Hz, 3 H); ^13^C NMR (50 MHz, CDCl_3_): δ 175.3, 171.9, 80.6, 70.4, 64.5, 34.4, 31.9, 31.7, 29.6, 29.5, 29.4, 29.3, 28.0, 24.7, 24.0, 22.6, 14.1; MS (m/z, ESI): [M + NH_4_]^+^ cald. for C_24_H_46_O_5_ 432.4; found, 432.3; analysis (calcd., found for C_24_H_46_O_5_): C (69.52, 69.36), H (11.18, 11.29).

### 4-(*tert*-Butoxy)-4-oxobutyl 6-(4-(hexyloxy)phenyl)-2-hydroxyhexanoate (15b)

Yield 35%; Yellowish oil, ^1^H NMR (200 MHz, CDCl_3_): δ 7.08 (d, *J* = 8.6 Hz, 2 H), 6.82 (d, J = 8.6 Hz, 2 H), 4.27–4.12 (m, 3 H), 3.93 (t, J = 6.0 Hz, 2 H), 2.78 (br s, 1 H), 2.58 (t, J = 7.0 Hz, 2 H), 2. 30 (t, J = 7.0 Hz, 2 H), 1.95 (q, J = 7.0 Hz, 2 H), 1.85–1.47 (m, 8 H), 1.46 (s, 9 H) 1.45–1.20 (m, 6 H), 0.91 (t, *J* = 7.0 Hz, 3 H); ^13^C NMR (50 MHz, CDCl_3_): δ 175.1, 172.8, 157.1, 134.1, 129.1, 114.2, 80.3, 70.3, 67.9, 64.4, 34.7, 34.2, 31.5, 31.3, 30.3, 29.2, 28.0, 25.7, 24.4, 23.6, 22.5, 14.0; MS (m/z, ESI): [M + NH_4_]^+^ calcd. for C_26_H_42_O_6_ 468.3; found, 468.1; analysis (calcd., found for C_26_H_42_O_6_): C (69.30, 69.08), H (9.40, 9.61).

### 4-(*tert*-Butoxy)-4-oxobutyl 2-hydroxy-6-phenylhexanoate (15c)

Yield 50%; Yellow oil, ^1^H NMR (200 MHz, CDCl_3_): δ 7.33–7.08 (m, 5 H), 4.23–4.10 (m, 3 H), 2.86 (br s, 1 H), 2.61 (t, J = 7.0 Hz, 2 H), 2.28 (t, J = 7.0 Hz, 2 H), 2.00–1.75 (m, 2 H), 1.70–1.45 (m, 6 H), 1.44 (s, 9 H); ^13^C NMR (50 MHz, CDCl_3_): δ 175.1, 162.8, 142.3, 128.3, 128.1, 125.6, 80.3, 70.3, 64.5, 36.5, 35.7, 34.2, 31.5, 28.0, 24.4, 23.9; MS (m/z, ESI): [M + NH_4_]^+^ calcd. for C_20_H_30_O_5_ 368.2; found, 368.3; analysis (calcd., found for C_20_H_30_O_5_): C (68.55, 68.34), H (8.63, 8.81).

### 4-*tert*-Butoxy-4-oxobutyl 6-(biphenyl-4-yl)-2-hydroxyhexanoate (15d)

Yield 61%; Oil, ^1^H NMR (200 MHz, CDCl_3_): δ 7.64–7.19 (m, 9 H), 4.41–4.00 (m, 3 H), 2.75 (br s, 1 H), 2.67 (t, J = 7.4 Hz, 2 H), 2.30 (t, J = 7.4 Hz, 2 H), 2.03–1.48 (m, 8 H), 1.45 (s, 9 H); ^13^C NMR (50 MHz, CDCl_3_): δ 175.1, 171.8, 141.4, 138.5, 129.5, 128.7, 128.6, 126.9, 126.8, 80.5, 70.2, 64.5, 35.3, 34.2, 31.6, 31.1, 28.0, 24.4, 23.9; MS (m/z, ESI): [M + Na]^+^ calcd. for C_26_H_34_O_5_ 449.2; found, 449.2; analysis (calcd., found for C_26_H_34_O_5_): C (73.21, 73.00), H (8.03, 8.21).

### 5-(*tert*-Butoxy)-5-oxopentyl 2-hydroxyhexadecanoate (15e)

Yield 48%; Light yellow oil; ^1^H NMR (200 MHz, CDCl_3_): δ 4.19–4.05 (m, 3 H), 2.84 (br s, 1 H), 2.21 (t, J = 6.7 Hz, 2 H), 1.70–1.45 (m, 8 H), 1.40 (s, 9 H), 1.30–1.15 (m, 22 H), 0.83 (t, J = 6.3 Hz, 3 H); ^13^C NMR (50 MHz, CDCl_3_): δ 175.6, 172.7, 80.5, 70.7, 65.3, 35.0, 34.6, 32.1, 29.9, 29.8, 29.7, 29.6, 29.5, 28.2, 28.1, 25.0, 22.9, 21.6, 14.3; MS (m/z, ESI): [M + NH_4_]^+^ calcd. for C_25_H_48_O_5_ 446.4; found, 446.3; analysis (calcd., found for C_25_H_48_O_5_): C (70.05, 69.89), H (11.29, 11.44).

### 5-(*tert*-Butoxy)-5-oxopentyl 6-([1,1′-biphenyl]-4-yl)-2-hydroxyhexanoate (15f)

Yield 70%; Colorless oil; ^1^H NMR (200 MHz, CDCl_3_): δ 7.64–7.14 (m, 9 H), 4.24–4.03 (m, 3 H), 2.70–2.54 (m, 3 H), 2.24 (t, J = 6.6 Hz, 2 H), 1.86–1.53 (m, 10 H), 1.44 (s, 9 H); ^13^C NMR (50 MHz, CDCl_3_): δ 175.3, 172.5, 141.5, 138.6, 129.5, 128.8, 128.7, 127.0, 126.9, 80.3, 70.3, 65.2, 35.4, 34.8, 34.3, 31.1, 28.1, 27.9, 24.5, 21.4; MS (m/z, ESI): [M + NH_4_]^+^ calcd. for C_27_H_36_O_5_ 458.3; found, 458.2; analysis (calcd., found for C_27_H_36_O_5_): C (73.61, 73.37), H (8.24, 8.39).

### 4-(*tert*-Butoxy)-4-oxobutyl 5-([1,1′-biphenyl]-4-yl)-2-hydroxypentanoate (15g)

Yield 26%; Colorless oil; ^1^H NMR (200 MHz, CDCl_3_): δ 7.64–7.20 (m, 9 H), 4.21 (m, 3 H), 2.85 (br s, 1 H), 2.69 (t, J = 7.0 Hz, 2 H), 2.29 (t, J = 7.2 Hz, 2 H), 2.06–1.66 (m, 6 H), 1.45 (s, 9 H); ^13^C NMR (50 MHz, CDCl_3_): δ 175.2, 172.6, 141.1, 139.2, 129.5, 128.8, 127.3, 127.2, 127.0, 80.8, 69.9, 64.8, 35.9, 34.8, 34.4, 28.4, 26.7, 21.5; MS (m/z, ESI): [M + NH_4_]^+^ calcd. for C_25_H_32_O_5_ 430.3; found, 430.3; analysis (calcd., found for C_25_H_32_O_5_): C (72.79, 72.60), H (7.82, 7.92).

### 5-(*tert*-Butoxy)-5-oxopentyl 5-([1,1′-biphenyl]-4-yl)-2-hydroxypentanoate (15h)

Yield 54%; Colorless oil; ^1^H NMR (200 MHz, CDCl_3_): δ 7.64–7.18 (m, 9 H), 4.30–4.08 (m, 3 H), 2.87 (br s, 1 H), 2.68 (t, J = 6.0 Hz, 2 H), 2.23 (t, J = 5.9 Hz, 2 H), 1.90–1.50 (m, 8 H), 1.44 (s, 9 H); ^13^C NMR (50 MHz, CDCl_3_): δ 175.5, 172.8, 141.3, 139.0, 129.0, 128.9, 127.3, 127.2, 127.1, 80.6, 70.5, 65.5, 35.3, 35.1, 34.2, 28.3, 28.1, 26.8, 21.7; MS (m/z, ESI): [M + NH_4_]^+^ calcd. for C_26_H_34_O_5_ 444.3; found, 444.3; analysis (calcd., found for C_26_H_34_O_5_): C (73.21, 73.07), H (8.03, 8.19).

### 4-Ethoxy-4-oxobutyl 2-hydroxy-6-phenylhexanoate (18)

Yield 57%; Yellow oil, ^1^H NMR (200 MHz, CDCl_3_): δ 7.30–7.05 (m, 5 H), 4.24–4.00 (m, 5 H), 2.92 (br s, 1 H), 2.61 (t, J = 7.1 Hz, 2 H), 2.36 (t, J = 6.0 Hz, 2 H), 1.96 (t, J = 7.1 Hz, 2 H), 1.80–1.60 (m, 4 H), 1.60–1.40 (m, 2 H), 1.24 (t, J = 6.0 Hz, 3 H); ^13^C NMR (50 MHz, CDCl_3_): δ 175.1, 172.5, 142.2, 128.2, 128.1, 125.6, 70.2, 64.4, 60.5, 35.6, 34.1, 31.0, 30.5, 24.4, 23.8, 14.1; MS (m/z, ESI): [M + NH_4_]^+^ calcd. for C_18_H_26_O_5_ 340.2; found, 340.3; analysis (calcd., found for C_18_H_26_O_5_): C (67.06, 66.93), H (8.13, 8.28).

### Synthesis of compounds 17a-h and 20

A solution of *tert*-butyl ester **16a-h** and **15a** (1 mmol) in 50% trifluoroacetic acid (TFA) in CH_2_Cl_2_ (10 mL) was stirred for 1 h at room temperature. The organic solvent was evaporated under reduced pressure and then CH_2_Cl_2_ was added and re-evaporated twice. The product was purified by precipitation with a mixture of EtOAc and petroleum ether (5:95, v/v, 10 mL) or by column chromatography (CH_2_Cl_2_-MeOH, 95:5).

### 4-((2-Oxohexadecanoyl)oxy)butanoic acid (17a, GK161)

Yield 85%; White solid; mp: 76–78 °C; ^1^H NMR (200 MHz, CDCl_3_): δ 9.25 (br s, 1 H), 4.32 (t, J = 6.0 Hz, 2 H), 2.82 (t, J = 6.0 Hz, 2 H), 2.51 (t, J = 6.0 Hz, 2 H), 2.15 (q, J = 6.0 Hz, 2 H), 1.80–1.50 (m, 2 H), 1.50–1.20 (m, 22 H), 0.88 (t, J = 7.0 Hz, 3 H); ^13^C NMR (50 MHz, CDCl_3_): δ 194.3, 178.6, 161.1, 65.0, 39.3, 31.9, 30.3, 29.6, 29.6, 29.4, 29.3, 29.3, 28.9, 23.4, 22.9, 22.7, 14.1; HRMS (m/z, ESI): [M-H]^−^ calcd. for C_20_H_36_O_5_ 355.2490; found, 355.2487; analysis (calcd., found for C_20_H_36_O_5_): C (67.38, 67.12), H (10.18, 10.39).

### 4-((6-(4-(Hexyloxy)phenyl)-2-oxohexanoyl)oxy)butanoic acid (17b, GK186)

Yield 54%; Low melting point white solid; ^1^H NMR (200 MHz, CDCl_3_): δ 9.20 (br s, 1 H), 7.04 (d, J = 8.6 Hz, 2 H), 6.77 (d, J = 8.6 Hz, 2 H), 4.26 (t, J = 7.0 Hz, 2 H), 3.89 (t, J = 7.0 Hz, 2 H), 2.81 (t, J = 7.0 Hz, 2 H), 2.60–2.45 (m, 2 H), 2.39 (t, J = 7.0 Hz, 2 H), 2.00 (q, J = 7.0 Hz, 2 H), 1.72 (t, J = 7.0 Hz, 2 H), 1.65–1.50 (m, 4 H), 1.48–1.35 (m, 2 H), 1.35–1.20 (m, 4 H), 0.86 (t, J = 7.0 Hz, 3 H); ^13^C NMR (50 MHz, CDCl_3_): δ 194.4, 178.1, 161.8, 157.1, 134.1, 129.1, 114.2, 67.9, 65.4, 34.7, 34.2, 31.5, 30.3, 29.2, 28.0, 25.7, 24.4, 23.6, 22.5, 14.0; MS (m/z, ESI): [M-H]^−^ calcd. for C_22_H_32_O_6_ 391.2; found, 391.4; HRMS (m/z, ESI): [M-H]^−^ calcd. for C_22_H_32_O_6_ 391.2126; found, 391.2122; analysis (calcd., found for C_22_H_32_O_6_): C (67.32, 67.13), H (8.22, 8.39).

### 4-((2-Oxo-6-phenylhexanoyl)oxy)butanoic acid (17c)

Yield 60%; Colorless oil; ^1^H NMR (200 MHz, CDCl_3_): δ 9.23 (br s, 1 H), 7.32–7.05 (m, 5 H), 4.29 (t, J = 6.0 Hz, 2 H), 2.84 (t, J = 8.0 Hz, 2 H), 2.63 (t, J = 8.0 Hz, 2 H), 2.48 (t, J = 6.0 Hz, 2 H), 2.06 (q, J = 8.0 Hz, 2 H), 1.75–1.55 (m, 4 H); ^13^C NMR (50 MHz, CDCl_3_): δ 194.3, 178.9, 161.3, 142.1, 128.6, 128.1, 126.1, 65.3, 39.3, 35.8, 30.8, 30.5, 23.6, 22.7; MS (m/z, ESI): [M + NH_4_]^+^ calcd. for C_16_H_20_O_5_ 310.2; found, 310.1; analysis (calcd., found for C_16_H_20_O_5_): C (65.74, 65.53), H (6.90, 7.08).

### 4-(6-(Biphenyl-4-yl)-2-oxohexanoyloxy)butanoic acid (17d, GK200)

Yield 94%; White solid; mp: 101–103 °C; ^1^H NMR (200 MHz, CDCl_3_): δ 9.25 (br s, 1 H),7.63–7.17 (m, 9 H), 4.37–4.21 (m, 2 H), 2.93–2.79 (m, 2 H), 2.75–2.58 (m, 2 H), 2.55–2.40 (m, 2 H), 2.14–1.95 (m, 2 H), 1.81–1.59 (m, 4 H); ^13^C NMR (50 MHz, CDCl_3_): δ 194.0, 178.7, 160.9, 141.0, 140.2, 138.7, 128.8, 128.7, 127.2, 127.0, 126.9, 65.0, 39.1, 35.1, 30.5, 30.2, 23.3, 22.5; MS (m/z, ESI): [M-H]^−^ calcd. for C_22_H_24_O_5_ 367.2; found, 367.3; HRMS (m/z, ESI): [M-H]^−^ calcd. for C_22_H_24_O_5_ 367.1551; found, 367.1544; analysis (calcd., found for C_22_H_24_O_5_): C (71.72, 71.49), H (6.57, 6.79).

### 5-((2-Oxohexadecanoyl)oxy)pentanoic acid (17e, GK433)

Yield 66%; White solid; mp: 77–79 °C; ^1^H NMR (200 MHz, CDCl_3_): δ 9.28 (br s, 1 H), 4.25 (t, J = 6.0 Hz, 2 H), 2.80 (t, J = 7.3 Hz, 2 H), 2.40 (t, J = 6.8 Hz, 2 H), 1.88–1.46 (m, 6 H), 1.34–1.15 (m, 22 H), 0.85 (t, J = 7.0 Hz, 3 H); ^13^C NMR (50 MHz, CDCl_3_): δ 194.8, 179.6, 161.4, 65.9, 39.6, 33.6, 32.1, 29.9, 29.8, 29.7, 29.6, 29.5, 29.2, 27.9, 23.2, 22.9, 21.2, 14.4; HRMS (m/z, ESI): [M-H]^−^ calcd. for C_21_H_38_O_5_ 369.2646; found, 369.2660; analysis (calcd., found for C_21_H_38_O_5_): C (68.07, 67.82), H (10.34, 10.52).

### 5-((6-([1,1′-Biphenyl]-4-yl)-2-oxohexanoyl)oxy)pentanoic acid (17f, GK452)

Yield 91%; White solid; mp: 83–85 °C; ^1^H NMR (200 MHz, CDCl_3_): δ 9.25 (bs, 1 H), 7.64–7.12 (m, 9 H), 4.25 (t, J = 7.0 Hz, 2 H), 2.87 (t, J = 6.0 Hz, 2 H), 2.67 (t, J = 5.9 Hz, 2 H), 2.40 (t, J = 6.3 Hz, 2 H), 1.89–1.54 (m, 8 H); ^13^C NMR (50 MHz, CDCl_3_): δ 194.2, 179.0, 161.1, 141.0, 138.7, 128.8, 128.7, 127.0, 126.9, 65.7, 39.1, 35.2, 33.2, 30.6, 27.6, 22.5, 20.9; MS (m/z, ESI): [M + NH_4_]^+^ calcd. for C_23_H_26_O_5_ 400.2; found, 400.2; HRMS (m/z, ESI): [M + Na]^+^ calcd. for C_23_H_26_O_5_ 405.1672; found, 405.1677; analysis (calcd., found for C_23_H_26_O_5_): C (72.23, 72.04), H (6.85, 6.99).

### 4-((5-([1,1′-Biphenyl]-4-yl)-2-oxopentanoyl)oxy)butanoic acid (17g, GK457)

Yield 47%; Light yellow solid; mp: 58–60 °C; ^1^H NMR (200 MHz, CDCl_3_): δ 10.06 (br s, 1 H), 7.66–7.18 (m, 9 H), 4.29 (t, J = 6.3 Hz, 2 H), 2.88 (t, J = 7.2 Hz, 2 H), 2.70 (t, J = 8.0 Hz, 2 H), 2.49 (t, J = 7.2 Hz, 2 H), 2.15–1.90 (m, 4 H); ^13^C NMR (50 MHz, CDCl_3_): δ 194.1, 178.2, 161.1, 141.2, 140.2, 139.3, 129.1, 128.9, 127.4, 127.3, 127.2, 65.3, 38.7, 34.5, 30.4, 24.6, 23.6; HRMS (m/z, ESI): [M + Na]^+^ calcd. for C_21_H_22_O_5_ 377.1359; found, 377.1357; analysis (calcd., found for C_21_H_22_O_5_): C (71.17, 71.02), H (6.26, 6.45).

### 5-((5-([1,1′-Biphenyl]-4-yl)-2-oxopentanoyl)oxy)pentanoic acid (17h, GK458)

Yield 65%; Light yellow solid; mp: 88–90 °C; ^1^H NMR (200 MHz, CDCl_3_): δ 9.81 (br s, 1 H), 7.65–7.18 (m, 9 H), 4.25 (t, J = 6.9 Hz, 2 H), 2.88 (t, J = 7.2 Hz, 2 H), 2.71 (t, J = 7.5 Hz, 2 H), 2.40 (t, J = 6.7 Hz, 2 H), 2.12–1.90 (m, 2 H), 1.81–1.65 (m, 4 H); ^13^C NMR (50 MHz, CDCl_3_): δ 194.4, 179.5, 161.3, 141.2, 140.5, 139.3, 129.1, 128.9, 127.4, 127.3, 127.2, 66.0, 38.8, 34.6, 33.6, 27.9, 24.7, 21.2. HRMS (m/z, ESI): [M + Na]^+^ calcd. for C_22_H_24_O_5_ 391.1516; found, 391.1504; analysis (calcd., found for C_22_H_24_O_5_): C (71.72, 71.48), H (6.57, 6.71).

### 4-((2-Hydroxyhexadecanoyl)oxy)butanoic acid (20, GK515)

Yield 85%; Low melting point white solid; ^1^H NMR (200 MHz, CDCl_3_): δ 9.26 (br s, 1 H), 4.30–4.10 (m, 3 H), 2.78 (br s, 1 H), 2.46 (t, J = 6.0 Hz, 2 H), 2.10–1.90 (m, 2 H), 1.85–1.50 (m, 4 H), 1.50–1.10 (m, 22 H), 0.87 (t, J = 7.0 Hz, 3 H); ^13^C NMR (50 MHz, CDCl_3_): δ 178.2, 175.4, 70.5, 64.4, 34.4, 31.9, 30.3, 29.7, 29.6, 29.5, 29.3, 24.8, 23.7, 22.7, 14.1; HRMS (m/z, ESI): [M-H]^−^ calcd. for C_20_H_38_O_5_ 357.2646; found, 357.2639; analysis (calcd., found for C_20_H_38_O_5_): C (67.00, 66.81), H (10.68, 10.89).

### *In vitro* PLA_2_ activity assay

The activities of human GVIA iPLA_2_, GIV cPLA_2_ and GV sPLA_2_ were determine using a group-specific mixed micelle modified Dole assay^27, 28, 30^. The substrate was prepared using slightly different conditions for each enzyme to achieve optimum activity: (i) GIVA cPLA_2_ mixed micelle substrate consisted of 400 μM Triton X-100, 95.3 μM PAPC, 1.7 μM arachidonyl-1-^14^C PAPC, and 3 μM phosphatidyl inositol (4,5)-bisphosphate (PIP_2_) in a buffer containing 100 mM 4-(2-hydroxyethyl)-1-piperazineethanesulfonic acid (HEPES) pH 7.5, 90 μM CaCl_2_, 2 mM dithiothreitol (DTT), and 0.1 mg/ml bovine serum albumin (BSA); (ii) GVIA iPLA_2_ mixed micelle substrate consisted of 400 μM Triton X-100, 98.3 μM 1-palmitoyl-2-arachidonylphosphatidylcholine (PAPC), and 1.7 μM arachidonyl-1-^14^C PAPC in a buffer containing 100 mM HEPES pH 7.5, 2 mM adenosine triphosphate (ATP), and 4 mM DTT; and (iii) GV sPLA_2_ mixed micelles substrate consisted of 400 μM Triton X-100, 98.3 μM PAPC, and 1.7 μM arachidonyl-1-^14^C PAPC in a buffer containing 50 mM tris(hydroxymethyl)aminomethane hydrochloride (Tris-HCl) pH 8.0, and 5 mM CaCl_2_. The compounds were initially screened at 0.091 mole fraction (5 μL of 5 mM inhibitor in dimethyl sulfoxide (DMSO)) in substrate (495 µL). *X*_I_(50) was determined for compounds exhibiting greater than 95% inhibition. Inhibition curves were generated using GraphPad Prism 5.0 and the non-linear regression by plotting percentage of inhibition vs log (mole fraction) to calculate the reported *X*_I_(50) and its associated error.

### Docking Calculations

Enzyme structures were optimized using the PPW. The structures of the inhibitors were sketched using Maestro sketcher and they were optimized using LigPrep. Glide was used for the rigid-docking of the compounds into the enzyme active site. The grid required for the docking procedure was generated using a scaling factor of 1.0 and partial charge cutoff of 0.25, while *X*, *Y*, *Z* dimensions of the inner box were set to 12 Å. For the inhibitor docking a scaling factor of 0.8 and partial charge cutoff of 0.15 were used that allow complete flexibility of the structures. The poses were selected according to the binding mode and the XP GScore. The Glide Extra-Precision (XP) scoring function was used for the calculations^63^.

### Macrophage Eicosanoid Production

RAW264.7 murine macrophage cells (ATCC #TIB-71) were maintained at 37 °C, 5% CO_2_ in DMEM (Life Technologies 11995–065) containing 10% FBS (Gemini), 100 U/mL penicillin/streptomycin, 1 mM sodium pyruvate and 4 mM L-glutamine. Macrophages were plated in 12-well tissue culture plates in 1 mL phenol red-free DMEM (Life Technologies) at a concentration of 5 × 10^5^ macrophages per well and were allowed to adhere for 24 hours. Wells receiving inhibitor treatment were spiked with **17f** to a final concentration of 5 μM and incubated for 1 hour at 37 °C. Kdo2-Lipid A (KLA; Avanti Polar Lipids) was then added to a final concentration of 100 ng/mL. Supernatants were collected at 24 hours for eicosanoid quantification. Cells were washed 2 times with 1 mL PBS and then collected in 1 mL PBS for determination of total protein concentration using a Pierce BCA assay kit (ThermoFisher). Supernatants and cellular material were stored at −80 °C until analysis. Supernatants were thawed on ice, and then spiked with 100 μL of an internal standard mix in ethanol (100 pg/μL; Cayman). Samples were purified via solid-phase extraction (SPE) and prepared for eicosanoid analysis as described in detail previously^64^. Briefly, following SPE, 10 μL of each sample was separated by reversed-phase liquid chromatography over 5.3 minutes using a gradient of the mobile phase A [water:acetonitrile:acetic acid (60:40:0.02; v/v/v)] and mobile phase B [acetonitrile:isopropanol (50:50; v/v)] on a 2.1 × 100 mm Acuity UPLC ® BEH Shield RP18 1.7 μm column. Online UPLC-electrospray ionization MS/MS quantitation of eicosanoids was performed on a QTRAP 6500 hybrid quadrupole/linear ion-trap mass spectrometer (AB Sciex) via multiple reaction monitoring (MRM) in negative ion mode. Eicosanoids were quantified by comparing the MRM signal and retention time to a pure standard. GraphPad Prism 7.0 was used for statistical analysis. Statistical significance was determined by one-way ANOVA analysis of variance and a Dunnett’s post-test comparing all columns to KLA treatment, P ≤ 0.05.
